# X-ray Single Crystal Structure, DFT Calculations and Biological Activity of 2-(3-Methyl-5-(pyridin-2’-yl)-1*H*-pyrazol-1-yl) Ethanol

**DOI:** 10.3390/molecules21081020

**Published:** 2016-08-05

**Authors:** Smaail Radi, Ahmed Attayibat, Mohamed El-Massaoudi, Amin Salhi, Driss Eddike, Monique Tillard, Yahia N. Mabkhot

**Affiliations:** 1 Laboratoire de Chimie Appliquée et Environnement (LCAE), Faculté des Sciences, Université Mohamed I, 60000 Oujda, Morocco; attayibat@yahoo.fr (A.A.); elmassaoudio@gmail.com (M.E.-M.); amiinsalhii@gmail.com (A.S.); 2 Laboratoire de Chimie du Solide Minéral et Analytique, Faculté des Sciences, Université Mohamed I, 60000 Oujda, Morocco; eddrisse@yahoo.fr; 3 Institut Charles Gerhardt-AIME, UMR 5253, CC1502, Université de Montpellier, 2 place Eugène Bataillon, 34095 Montpellier Cedex 5, France; 4 Department of Chemistry, Faculty of Science, King Saud University, P.O. Box 2455, 11451 Riyadh, Saudi Arabia; yahia@ksu.edu.sa

**Keywords:** pyridylpyrazole, single-crystal, DFT calculations, reactivity indices, Fukui functions, Parr functions, biological activity

## Abstract

A pyridylpyrazole bearing a hydroxyethyl substituent group has been synthesized by condensation of (*Z*)-4-hydroxy-4-(pyridin-2-yl)but-3-en-2-one with 2-hydroxyethylhydrazine. The compound was well characterized and its structure confirmed by single crystal X-ray diffraction. Density functional calculations have been performed using DFT method with 6-31G* basis set. The HOMO-LUMO energy gap, binding energies and electron deformation densities are calculated at the DFT (BLYP, PW91, PWC) level. The electrophilic f(−) and nucleophilic f(+) Fukui functions and also the electrophilic and nucleophilic Parr functions are well adapted to find the electrophile and nucleophile centers in the molecule. The title compound has been tested for its DPPH radical scavenging activity which is involved in aging processes, anti-inflammatory, anticancer and wound healing activity. Compound is also found with a significant antioxidant activity, probably due to the ability to donate a hydrogen atom to the DPPH radical.

## 1. Introduction

Pyrazole rings have been described extensively in the literature as chelating ligands [1,2,3,4,5,6], several reports being reviews [7,8,9]. These derivatives are also well established in the literature as important biologically active heterocyclic compounds, such as antitumor [10], antiviral [11], anti-inflammatory [12] anti-anxiety [13] and antimicrobial [14] agents.

Pyrazoles associated with pyridine groups show higher chelation ability [15,16,17,18]. This aptitude is mainly due to the presence of diversified sp^2^ hybrid nitrogen donors with the involvement of geometry and the nature of the ligands. This chelating activity is accompanied by efficient biological activity of these drugs as insecticides [19] and fungicides [20]. On the other hand, compounds with hydroxyl substituents are known to show good pharmaceutics [21,22] and antimicrobial properties [23]. Indeed, the presence of hydroxyl groups on the aromatic ring make these products antioxidants that can scavenge free radicals. The hydroxyl radical is an extremely reactive oxidizing radical that can react with most biomolecules, including proteins, lipids and DNA in its vicinity at controlled diffusion rates. They can be produced in vivo by the homolytic breakage of oxygen-hydrogen bonds in water driven by the continuous exposure to background ionizing radiation.

Taken together, all this information suggests that pyridylpyrazoles bearing hydroxyl substituents may be a key moiety in the treatment of diseases related to free-radical damage. However, there are only a few studies concerning pyridylpyrazole compounds. It is therefore interesting to increase the diversity of compounds containing these efficient and versatile ligands. Herein, we report on the synthesis of a pyridylpyrazole derivative with a hydroxyl substituent in the side chain. Its X-ray single crystal structure was determined and DFT calculations are reported. The radical scavenging and antioxidant activities of the compound were also tested.

## 2. Results

### 2.1. Chemistry

**Scheme 1:**
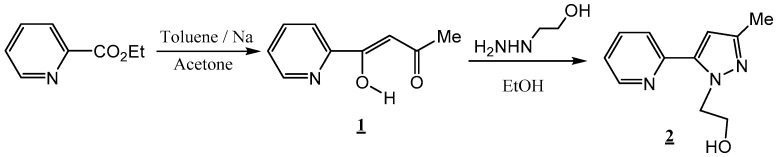
Synthesis of the target compound.

The target compound based on a pyridylpyrazole core with a hydroxyl-functionalised arm was prepared in two steps (Scheme 1). The first synthesis step is the preparation of (2*Z*)-3-hydroxy-1-(pyridin-2-yl)but-2-en-1-one ligand **1** [24]. The reaction was carried out using ethyl 2-pyridinecarboxylate and acetone as nucleophile under mild Claisen condensation conditions (room temperature, two days), using toluene as solvent and sodium metal as the base. This procedure afforded exclusively the target product in its enol tautomeric form.

The second step involves condensation of the ligand **1** with 2-hydroxyethylhydrazine using our previously described method [25]. This reaction affords the desired compound **2** in 54% yield as the major product. All the structures are in perfect agreement with their spectroscopic and analytical data.

### 2.2. X-ray Crystal Structure Description

The title compound 2-(3-methyl-5-(pyridin-2′-yl)-1*H*-pyrazol-1-yl) ethanol was subjected to X-ray diffraction analysis. Crystal data and refinement parameters are listed in Table 1. Supplementary data are deposited at CCDC under deposition number 1487543.

**Figure 1:**
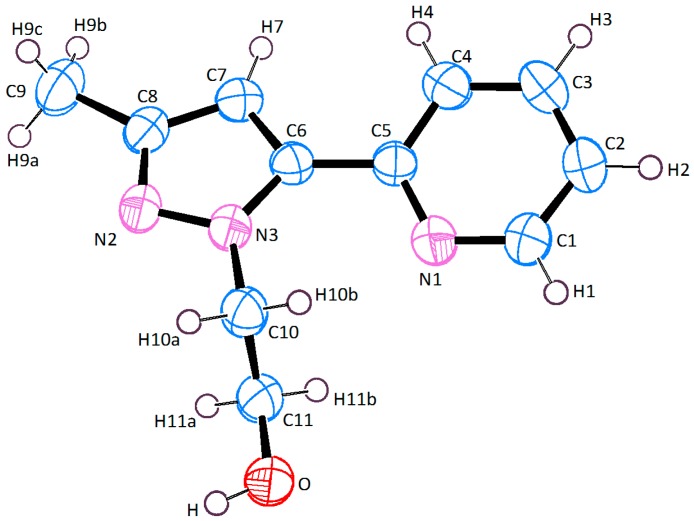
Asymmetric unit of C_11_H_13_N_3_O.

Each molecule of the title compound is constituted of two rings (Figure 1) a pyridyl bonded by a 1.472(3)Å C-C junction to a pyrazole substituted by methyl and ethanol groups in the β-position. These two rings are essentially planar, as reflected by the rms deviations of fitted atoms, that are respectively 0.0075 and 0.0054Å, and they are nearly coplanar as indicated by the dihedral angle value of 10.65(2)°. The bond lengths and angles between atoms in each cycle are comparable to those found in the literature [26].

**Figure 2:**
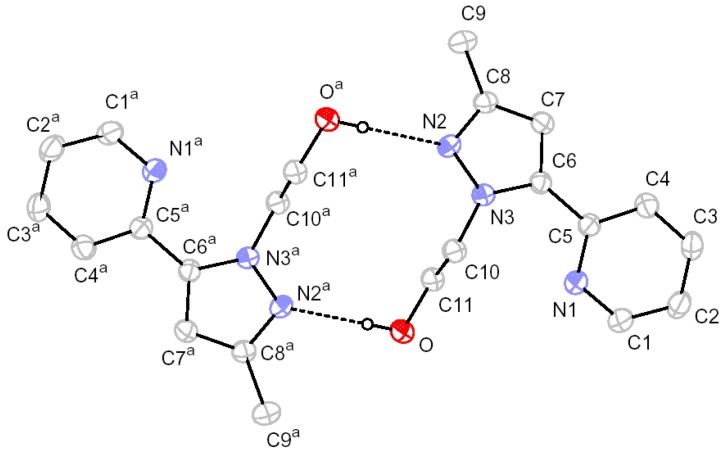
Molecular structure of **2**, thermal ellipsoids are represented at 30% probability. Hydrogen atoms (except that bonded to O atom) have been omitted for clarity. Symmetry codes used to generate equivalent atoms: − x + 0.5, − y + 0.5, − z (a).

**Figure 3:**
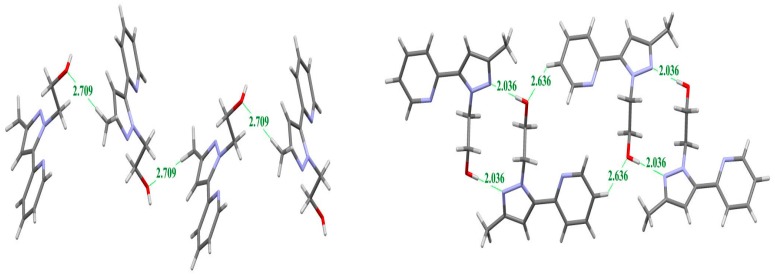
Representation of the molecular arrangement enhancing the intermolecular interactions viewed along a-axis (**left**) and b-axis (**right**). Atoms colours codes: O (red), N (blue), C (grey) and H (white).

Molecules are pairwise linked through N2···H–O hydrogen bonds of 2.855(2)Å length into centrosymmetric dimers (N2···H–O angle 179.0°) shown in Figure 2. In each dimer, the cycles are arranged parallel to the (101) plane. The dimers are also involved with homologous units in weak intermolecular van der Waals interactions of 2.636Å (O···H2) and 2.709Å (O···H9b) as represented in Figure 3. In addition to the short hydrogen bonds N2–O that participate in dimer formation, some weaker interactions occur between dimers. Each dimer is further involved in interactions with two neighbours through N2-C7 contacts of 3.746(2)Å (N2···H7–C7 172.4°) and also with four additional molecules through contacts that involve the oxygen atom at the end of the ethyl branch. The distances are respectively 3.327(2) and 3.635(3)Å for the O-C9 and O-C2 contacts and angles are 131.7° and 162.4° for O···H9-C9 and O··H2-C2. On the other hand, each N1 atom participates in intramolecular interaction with C10, (N1-C10 is 2.919(2)Å and N1···H10-C10 is 114.4°). All these intermolecular interactions that result from molecular packing in the unit cell contribute to stabilization of the compound in its solid state.

### 2.3. DFT Calculations

Density functional theory (DFT) has been used to investigate the molecular geometry and the electron distribution. The geometric configurations have been optimized using the program DMol3. Results are obtained at different DFT levels within the GGA generalized gradient approximation with BLYP or PW91 functional as well as within the LDA local density approximation with PWC functional [27,28,29]. In all cases DNP double numerical plus polarization basis set have been used.

**Figure 4:**
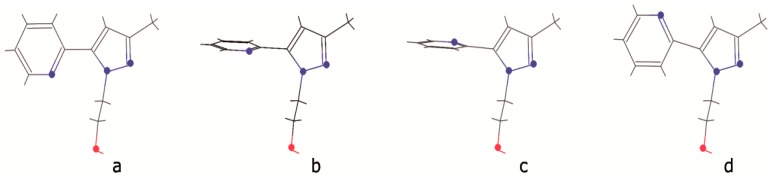
Four different conformations of the studied molecule (**a**–**d**) differing by the orientation of the pyridine cycle. Oxygen and nitrogen atoms are represented as red and blue points.

Full geometry optimizations by minimization of the total energy have been carried out, starting from four different conformations of the molecule. While conformers b and c (Figure 4), with the pyridine ring rotated by 90° from its position, revert into the experimental conformation a, the rotamer d (180°-rotation of pyridine ring) retains its initial geometry.

Nevertheless, the calculated energy is higher by ~3 kcal/mol than that of experimental conformation, indicating a lower stability. This should be related to the intramolecular interaction of van der Waals type between C10 and N1 atoms that occur in the experimental conformation.

Geometry has been optimized for an individual molecule and for a dimer considering molecular fragments isolated from the crystal structure as starting models. Similar calculations considering the complete unit cell packing have been performed to describe the crystalline solid state. Bond length, bond and torsion angles are of interest for the structure analysis. Some selected experimental parameters are given in Table 2 together with the calculated values for the molecule, dimer and crystalline state. Their variations give an evaluation of the structural packing constraints on the molecular geometry.

Particularly interesting are the respective positions within a molecule of the pyridine and pyrazole rings. We note that these aromatic rings are not strictly coplanar and deviation from flatness can be measured by the dihedral angle between their mean planes. Values calculated for the corresponding N1–C5–C6–N3 angle are quite different in the molecule and in the crystal. In the meantime the rather long N1–N3 intramolecular distance is significantly affected by crystal packing. For example, in the GGA PW91 calculation, the dihedral angle is reduced by hydrogen bonding dimerization from 20.47 to 16.86° and then reaches the experimental value of 11.8° in solid state while the N–N distance is notably shortened (from 2.948 to 2.931 Å).

It can be concluded that molecular packing in the solid state causes a flattening of the molecule that would allow conjugation effects to be extended. Nevertheless, the experimental C5-C6 bond length of 1.472 Å, slightly longer than computed distances, is of the order of those observed in similar compounds and does not provide much information about inter-ring conjugation.

According to the FMO theory, the form and position of frontier orbitals are relevant for the reactivity of a molecule [30]. Whilst the lowest empty LUMO is highly π*-antibonding at the two cycles, the highest filled HOMO displays a π-bonding mainly localized at the pyrazole ring. From all calculations (molecule, dimer or solid state) at the different DFT levels of theory, these orbitals are found separated by an energy gap of 3.4 eV which is to compare with the experimental value of 4.06 eV from UV experiments (absorption peak at 304 nm).

**Figure 5:**
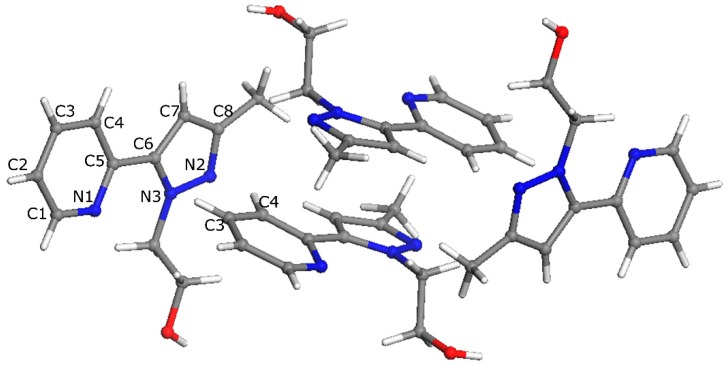
Molecular stacking representation emphasizing π/π interactions between molecules. Atoms colours codes: O (red), N (blue), C (grey) and H (white).

On the other hand, a particular stacking is observed along the [hkl] direction with the pyridine ring of a molecule superimposed with the pyrazole ring of a neighbouring molecule and vice versa. In addition to these face-to-face (parallel) π/π interactions, edge-to-face (T-shape) π/π interaction occur between a pyridine ring and pyrazole ring of a neighbouring molecule as represented in Figure 5.

**Figure 6:**
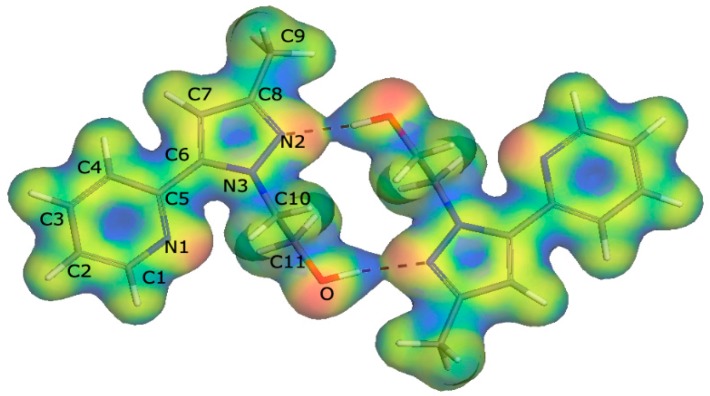
Representation of the isosurface electron density (3D volumic contour) mapped with the deformation density (red color indicates electron localisation. Bule color indicates out electron losses, whereas the green/yellow represents a potential halfway between the two extremes.

The plane-to-plane distance between two neighbouring molecules is about 3.5Å, but no bonding density can be evidenced in this region. Instead significant densities are computed at N2-O atomic pairs involved in hydrogen bonding and very slight densities at C10-N1 and C2-O atomic pairs involved in intermolecular van der Waals interactions. This can be visualized with the 3D contour of the total density represented in Figure 6 at the 0.15 iso-level.

The deformation density, which is the total density with the density of isolated atoms subtracted, is mapped on the isosurface total density and shows high positive values (red) indicative of electron localization while low negative values (blue) point out electron losses. Looking at this representation (Figure 6), in addition to high bonding populations at intramolecular bonds and at the intermolecular hydrogen bonding, it is evident that the highest values are calculated at electronegative atoms and are rather non-bonding densities.

Deformation densities and molecular electrostatic potential have been shown to be strongly related and both may be used to discuss the reactivity of compound [31]. The calculated electrostatic potential found over the whole molecule would be a good tool to evaluate the regiochemistry especially for reactions dominated by electrostatic effects.

More informative are the Fukui indices which are computed from the electronic density, and give a measurement of the local reactivity. The higher the Fukui indices, the higher the reactivity. The Fukui functions are defined as derivatives of the electron density with respect to the number of electrons at a constant potential. Then for an optimized geometry, changes in the calculated density when adding or removing an electron will point out the reactive regions of the molecule. The electrophilic f(−) and nucleophilic f(+) Fukui functions can be condensed to the nuclei by the use of a partitioning scheme of the atomic charge as Mulliken [32] or Hirshfeld [33].

**Figure 7:**
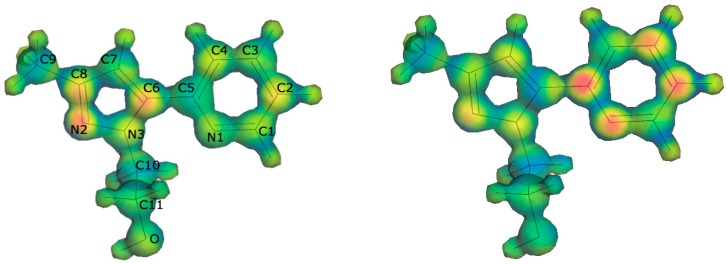
Representation of the Fukui functions projected onto the electrostatic potential (Higher values correspond to red zones). Left: electrophilic f(−) and right: nucleophilic f(+). Lower values correspond to blue zones, whereas the green/yellow represents a potential halfway between the two extremes

Directly related to the electrophilicity and nucleophilicity, their high values reflect the susceptibility for an electrophilic or nucleophilic attack. Several recent studies have proved the relevance of such analyses and successfully used to rationalize the regioselectivity [34,35,36,37]. An easy graphical view of the molecule regioselectivity is provided in Figure 7 by the representation of f(−) and f(+) Fukui functions projected onto the molecular electrostatic potential. Atoms with high f(−) values are most likely to suffer an electrophilic attack as N2 atom at pyrazole ring which is also the best place for a radical attack as indicated by the f(0) function (not represented).

Nevertheless, some recent works devoted to establish the regioselectivity in polar reactions report that the use of Fukui functions is not a good choice and that local electrophilicity may constitute an improved alternative for reactivity description [38]. On the other hand, it has been established [39,40] that the electrophilic and nucleophilic Parr functions are well adapted to find the electrophile and nucleophile centers in a molecule. These functions, respectively P_k_^+^ and P_k_^−^, are considered as powerful tools for the study of organic reactivity. They can be obtained from the analysis of atomic spin density of the radical anion and the radical cation. Thus, spin unrestricted calculations have been performed for the molecule (in its optimized neutral geometry) either bearing a +1 or −1 charge in order to compute the spin densities (difference between α and β electron densities) at each atom. The atomic spin density spatial distribution can be visualized as an isosurface 3D contour and also by checking the calculated values at each atom. The greatest values of Mulliken and Hirshfeld atomic spin densities are reported in Table 3 with the graphic representation at the 0.5 level. Results show the highest electrophilic area is located around C5 and N1 atoms while the highest nucleophilic center is found at N2 atom. In the present case, the Parr functions quite well corroborate the results predicted on the basis of Fukui functions.

The nucleophilic character of the N2 pyrazolic atom and its reactivity is justified by formation of a strong N2··· HO (dimer) hydrogen bond. Instead, atoms characterized with a high P_k_^+^ value are susceptible to undergo a nucleophilic attack; in the molecule these are mainly located in the pyridine ring (C5, N1, C2, C3).

The strength of all the intermolecular interactions involved in the solid state packing can be evaluated using binding energy. This energy associated to the structure cohesion corresponds to the energy needed to dissociate the molecule or the crystal into atoms at infinite separation. The strength of hydrogen bonds can be evaluated from comparison of binding energies calculated for the molecule and for the dimer. Since binding energy is found to increase from the molecule to crystal, it can be concluded that crystal packing is stabilized by the N2-HO hydrogen bonding and by the C10–N1 and C2–O van der Waals interactions.

### 2.4. Biological Activity

#### 2.4.1. DPPH Radical Scavenging Activity

In the present study, the possible radical scavenging activity of the pyridylpyrazole derivative was examined. It is known that compounds that include hydroxy substituents represent a significant source of reducers able to provide an electron or hydrogen radical to stabilize the 1,1-diphenyl-2-picrylhydrazyl (DPPH) radical in solution. In this case, the radical-scavenging activity has been studied by measuring the decrease in absorbance, in order to assess the capacity of the studied organic compound.

All results are expressed for each sample as a% activity related to BHA (a mixture of: 2-*tert*-butyl-4-hydroxyanisole and 3-*tert*-butyl-4-hydroxyanisole) and ascorbic acid references, which are strong DPPH radical scavengers. Table 4 reports the absorbance of DPPH for compound **2** which was characterized by a moderate radical scavenging activity compared to the standards.

As shown, at a concentration of 100 ppm, the compound exhibits a significant radical scavenging activity of about 73%. This considerable activity should probably be attributed to the high radical-scavenging property of the hydroxyl substituent, and is promising for the treatment of diseases caused by free radicals, and it provides more information for designing novel drugs.

**Figure 8:**
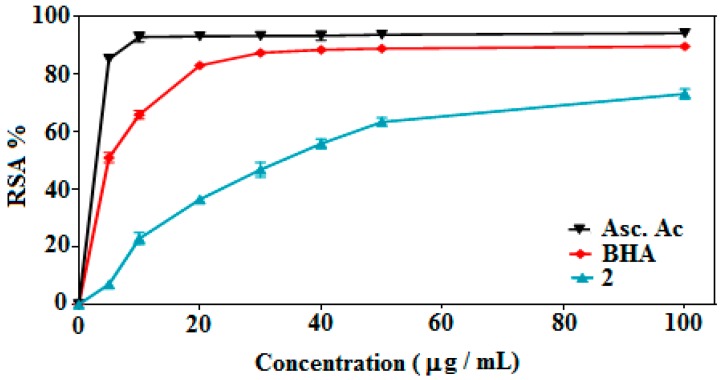
DPPH radical scavenging activity of compound **2**.

Moreover, the IC_50_ value in antioxidant assays was calculated from the plotted curves using regression analyses (Figure 8). The result show moderate antioxidant activity (IC_50_ value 31.8 µg/mL) compared to the ascorbic acid (IC_50_ value 2.82 µg/mL) and BHA (IC_50_ value 6.8 µg/mL) standards.

#### 2.4.2. Ferric Reducing Antioxidant Power (FRAP) Assay 

**Figure 9:**
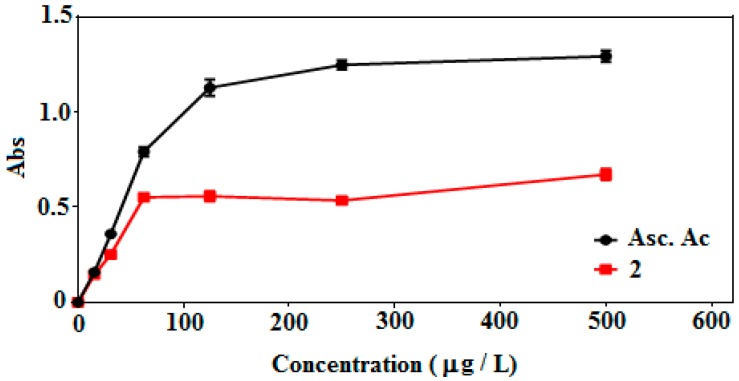
Reducing power activity of compound **2**.

The FRAP assay is a simple, inexpensive and reproducible process based on the ability of a sample to reduce the ferric Fe^3+^ to ferrous Fe^2+^ ion. Therefore, the ability of a compound to transfer an electron is a significant indicator of its antioxidant potentialities [41]. Figure 9 shows the optical density (OD) read at 700 nm according to the sample concentration and with ascorbic acid taken as standard. Indeed, the reducing power of both sample and standard increases with the concentration.

## 3. Experimental Section

### 3.1. General Information

All solvents and other chemicals (purity > 99.5%, Aldrich, Saint-Louis, MO, USA) of analytical grade were used without further purification. An Xcalibur CCD diffractometer (Oxford Diffraction, Abingdon, Oxfordshire, UK) was used to perform X-ray analysis on a parallelepiped colourless sample. Elemental analyses were performed by the Microanalysis Centre Service (CNRS, Lille, France). Melting points were measured using a Büchi 510 m.p. apparatus (LCAE, Oujda, Morocco). ^1^H- and ^13^C-NMR spectra were recorded on an AC 300 spectrometer (CNRST) (Bruker, Rabat, Morocco) (300 MHz for ^1^H and 75.47 MHz for ^13^C spectra). A JMS DX-300 mass spectrometer (JEOL, Rabat, Morocco) was used for the determination of molecular weights. Infrared (IR) spectra were recorded on a Shimadzu infrared spectrophotometer (LCAE, Oujda, Morocco) using the KBr disc technique.

### 3.2. Synthesis of 2-(3-Methyl-5-(pyridin-2-yl)-1H-pyrazol-1-yl)ethan-1-ol (***2***)

To a solution of 1-pyridin-2-yl-butane-1,3-dione (**1**, 1.5 g, 9.2 × 10^−3^ mol) in absolute ethanol (50 mL) and cooled at 0 °C, was slowly added a solution of 2-hydroxyethylhydrazine (0.7 g, 9.2 × 10^−3^ mol) in absolute ethanol (10 mL). The mixture was stirred at room temperature for 2 h. Then, the solvent was removed under reduced pressure and the obtained residue was purified on silica gel using (20% ethanol/80% ether) to give 54% yield of **2** as a brown solid. The obtained solid was recrystallized using methanol to give colourless crystals of **2** suitable for X-ray analysis. Mp = 90–93 °C (Ether); ^1^H-NMR (CDCl_3_, 25 °C): δ = 2.34 (s, 3 H, CH_3_), 4.12 (t, *J*_H,H_ = 4.86 Hz and 5.40 Hz, 2 H, -CH_2_OH), 4.59 (t, *J*_H,H_ = 4.86 Hz and 5.40 Hz, 2H, N-CH_2_), 6.37 (s, 1H, pz-H), 7.32 (m, 1H, H_β_), 7.60 (d, 1H, H_δ_), 7.82 (t, 1H, H_γ_), 8.62 (d, 1H, H_α_) ppm; ^13^C-NMR (CDCl_3_, 25 °C): δ = 13.48 (-CH_3_), 52.29 (N-CH_2_-), 62.91 (-CH_2_-OH), 106.37 (Pz C-H), 122.94 (Py C-H_β_), 123.62 (Py C-H_δ_), 137.73 (Py C-H_γ_), 142.22 (Pz C), 148.05 (Pz CCH_3_), 148.31 (Py C-H_α_), 148.95 (Py C) ppm. Anal. Calc. for C_11_H_13_N_3_O: C 65.02, H 6.40, N 20.69. Found: C 65.00, H 6.39, N 20.74; *m*/*z*: 203 (M^+^). IR (KBr): ν(OH) = 3260 cm^−1^.

### 3.3. X-ray Crystallographic Analysis 

A single crystal of the title compound was selected for X-ray structural analysis and mounted on an Oxford Diffraction XCalibur CCD diffractometer [41] using Mo-Kα radiation (λ = 0.71073 Å). Unit cell dimensions with estimated standard deviations were determined by least-squares from the whole reflexion data set. A total of 15317 reflexions has been collected in θ range 3.30–26.14°, from which 2114 are independent and 1616 satisfy the intensity criterion I > 2σ(I). The intensities were corrected for Lorentz and polarisation effects. Main crystal data collection and refinement parameters are listed in Table 1. Data reduction was carried out using the Oxford Diffraction CrysAlis Red 171 [42] program.

The structure was solved by direct methods in the monoclinic space group C2/c and refined by full-matrix least-squares using SHELXS-97 and SHELXL-97 program packages [43]. The weighting scheme employed was w = 1/[σ^2^(F_o_^2^) + (0.0882P)^2^ + 0.3970P] where P = (F_o_^2^ + 2F_c_^2^)/3. Except for the hydrogen atom of OH group which was detected in the Fourier difference and freely refined, the H atoms were included at calculated positions and refined in riding mode. Molecular graphics are drawn with ORTEP-3 for windows [44] and Mercury 3.8. The complete crystallographic data have been deposited (CCDC-1487543) and can be obtained free of charge from the Cambridge Crystallographic Data Centre via http://www.ccdc.cam.ac.uk/data_request/cif.

### 3.4. DPPH Radical Scavenging Activity

The radical scavenging activity against stable 1,1-diphenyl-2-picryl hydrazyl (DPPH) radical has been examined using the method reported by Moure et al. [45] for the compound comparatively with activities of known antioxidant agents, BHA and ascorbic acid. Briefly 0.1 mL of sample (tested at concentrations ranging from 5 to 100 µg/mL) was added to 1.9 mL ethanol solution of the DPPH radical. After 30 min of incubation in the dark at room temperature, the absorbance was read against a blank at 517 nm. The scavenging activity DPPH radical was expressed as a percentage inhibition by the following formula:

[(*A_blank_* − *A_sample_*)/*A_blank_*] × 100 (1)

where, *A_sample_* is the absorbance of the solution containing the sample and *A_blank_* is the absorbance of the DPPH solution. The IC_50_ values were calculated as the concentration of extract causing a 50% inhibition of DPPH radical.

### 3.5. Ferric Reducing Antioxidant Power (FRAP) Assay 

Ferric reducing antioxidant power was determined according to the method described by Oyaizu (1986) [46]. This method may evidence the antioxidant behavior of reductants as they cause the reduction of [Fe(CN)_6_]^3−^ complex into [Fe(CN)_6_]^4−^. Actually, 2.5 mL of aqueous sample (7.8–500 μg/mL) are mixed with 2.5 mL of the buffer solution phosphate (0.2 M; pH 6.6) and 2.5 mL of potassium ferricyanide (1.0%) and mixture is incubated at 50 °C for 30 min. Thereafter, 2.5 mL of trichloroacetic acid (10%) is added. The whole is centrifuged at 3000 rpm for 10 min. For each concentration, 2.5 mL of the supernatant is mixed with 2.5 mL of distilled water and 0.5 mL FeCl_3_ (0.1%). Absorbance is measured at 700 nm using a spectrophotometer. The higher absorbance of the solution indicate the greater reducing power for the compound. For comparison, ascorbic acid has been used as positive control.

## 4. Conclusions

Based on the experimental results, a pyridylpyrazole derivative bearing a hydroxyl substituted arm group has been synthesized and its XRD single crystal structure determined. Density functional calculations have been performed using the DFT method. The HOMO-LUMO energy gaps, binding energies and molecular electrostatic potential (MEP) are calculated. In the present case, the Parr functions quite well corroborate the results predicted on the basis of Fukui functions. The title compound has been tested for its DPPH radical scavenging and antioxidant activities. Results show significant activities that are most probably due to the compound ability to donate a hydrogen atom to the DPPH radical.

## Figures and Tables

**Table 1:** Crystal data and refinement parameters for C_11_H_13_N_3_O.

CCDC Deposition Number	1487543
Molecular Formula	C_11_H_13_N_3_O
Molecular Weight	203.11
Crystal System	Monoclinic
Space Group	C2/c
*a* (Å)	18.837(2)
*b* (Å)	12.344(2)
*c* (Å)	9.3011(10)
α (°)	90
β (°)	101.939(10)
γ (°)	90
*V* (Å^3^)	2116.0(5)
*Z*	8
*D*calc (g·cm^−3^)	1.276
Crystal Dimension (mm)	0.17 × 0.28 × 0.34
μ (mm^−1^)	0.085
*T*min/*T*max	0.972/0.986
Measured Reflections	15317
	−24 ,24
Indices Range (*h*, *k*, *l*)	−16, 16
	12, 12
θ Limit (°)	3.30–26.14
Unique Reflections	2114
Observed Reflections (*I* > 2σ(*I*))	1616
Parameters	136
Goodness of Fit on *F*^2^	1.033
*R*_1_,*wR*_2_ [*I* > 2σ(*I*)]	0.0498, 0.1363

**Table 2:** Experimental and calculated distances (Å) and angles (°) for molecule, dimer and crystalline structure. Calculated energies are also given.

	RX		Molecule			Dimer			Solid	
	exp	BLYP	PW91	PWC	BLYP	PW91	PWC	BLYP	PW91	PWC
C8-N2	1.330	1.347	1.343	1.334	1.349	1.344	1.334	1.349	1.346	1.338
C1-N1	1.331	1.345	1.339	1.328	1.344	1.338	1.327	1.344	1.339	1.331
N1-C5	1.338	1.357	1.351	1.338	1.356	1.349	1.337	1.356	1.349	1.456
C5-C6	1.472	1.470	1.462	1.447	1.472	1.463	1.449	1.471	1.466	1.340
N3-C6	1.359	1.383	1.375	1.362	1.381	1.372	1.359	1.379	1.371	1.360
N3-N2	1.358	1.367	1.352	1.333	1.369	1.354	1.334	1.370	1.357	1.341
N1-N3	2.910	2.965	2.948	2.893	2.964	2.941	2.883	2.935	2.931	2.919
C10-C11	1.506	1.540	1.530	1.510	1.545	1.536	1.516	1.537	1.530	1.518
C11-O	1.411	1.444	1.429	1.405	1.430	1.415	1.390	1.433	1.419	1.399
N3-C10	1.459	1.472	1.459	1.438	1.476	1.462	1.441	1.471	1.460	1.441
C6-C5-N1	118.38	118.630	118.660	118.790	118.730	118.760	118.670	118.710	118.840	118.990
C5-C6-N3	125.66	126.13	126.04	125.48	126.19	126.06	125.24	125.67	125.84	125.88
C4-C5-C6	119.80	119.98	119.75	119.66	119.81	119.64	119.66	119.62	119.34	119.28
C4-C5-N1	121.80	121.41	121.57	121.54	121.41	121.59	121.67	121.66	121.81	121.73
C7-C6-N3	105.93	105.54	105.52	105.63	105.90	105.91	106.17	105.90	105.87	106.03
C6-N3-C10	131.12	131.09	130.57	129.98	131.30	130.88	130.56	130.90	130.52	130.08
C11-C10-N3	111.50	112.00	111.82	111.33	112.59	112.43	111.91	111.91	111.72	111.70
O-C11-C10	111.03	111.08	111.23	111.56	110.74	110.90	110.96	111.04	111.41	111.69
C4-C5-C6-N3	169.13	160.52	160.42	168.07	162.30	163.80	170.12	169.73	169.00	165.83
N3-C10-C11-O	177.15	176.40	176.30	174.34	174.52	174.60	174.08	178.46	177.17	176.39
N1-C5-C6-N3	11.80	20.44	20.47	12.27	18.39	16.86	10.29	10.92	11.8	14.92
C10-N1 (intra)	2.919	3.006	2.974	2.869	3.002	2.960	2.866	2.939	2.927	2.909
intramolecular	2.382	2.455	2.420	2.334	2.452	2.409	2.328	2.360	2.330	2.270
C10-H10-N1	114.35	109.84	109.83	107.84	109.74	109.64	108.04	111.42	112.47	114.81
O-N2 (inter)	2.855				2.927	2.862	2.703	2.840	2.817	2.760
intermolecular	2.036				1.941	1.875	1.700	1.846	1.823	1.751
O-H-N2	178.99				173.02	173.74	174.67	177.11	175.76	175.65
C2-O (inter)	3.327							3.242	3.278	3.314
intermolecular	2.636							2.405	2.429	2.410
C2-H2-O	131.63							132.93	133.87	137.64
E_tot_/molecule (Ha)		666.46	666.44	660.95	666.44	666.48	660.98	666.47	666.49	661.02
Binding E (kcal/mol)		3061.86	3187.32	3557.69	3068.01	3195.53	3571.68	3063.73	3202.79	3596.28

**Table 3:**
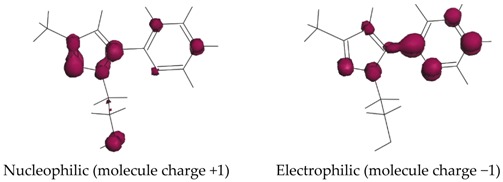
Calculated Mulliken and Hirshfeld atomic spin densities (Mulliken atomic spin density is represented at 0.5 level).

Atom	Mulliken	Hirshfeld	Mulliken	Hirshfeld
C8	0.108	0.092		
C6	0.166	0.126		
C5			0.235	0.174
C4	0.108	0.078		
C3			0.122	0.102
C2	0.136	0.097	0.238	0.160
N1			0.163	0.149
N3	0.084	0.094	0.094	0.088
N2	0.197	0.177	0.113	0.096
O	0.129	0.114		

**Table 4:** Radical scavenging activity of **2**.

Samples	Scavenging Ability (%, Mean ± SD), Concentration (µg/mL)						
	5.0	10.0	20.0	30.0	40.0	50.0	100.0
**2**	6.9 ± 1.4	22.8 ± 1.9	36.5 ± 0.8	46.8 ± 2.5	55.9 ± 1.6	63.4 ± 1.4	73.2 ± 1.7
**BHA**	51.0 ± 1.6	65.9 ± 1.5	83.1 ± 0.9	87.5 ± 0.1	88.5 ± 1.0	89.0 ± 1.2	89.7 ± 0.7
**Asc. acid**	85.3 ± 1.3	93.0 ± 1.7	93.2 ± 1.2	93.4 ± 1.1	93.4 ± 1.5	93.8 ± 1.0	94.3 ± 1.0
